# Steric and Slippage Effects on Mass Transport by Using an Oscillatory Electroosmotic Flow of Power-Law Fluids

**DOI:** 10.3390/mi12050539

**Published:** 2021-05-10

**Authors:** Ruben Baños, José Arcos, Oscar Bautista, Federico Méndez

**Affiliations:** 1 Instituto Politécnico Nacional, ESIME Azcapotzalco, Av. de las Granjas No. 682, Col. Santa Catarina, Del. Azcapotzalco, Ciudad de México 02250, Mexico; rbanosm1200@alumno.ipn.mx; 2 Departamento de Termofluidos, Facultad de Ingeniería, UNAM, Ciudad de México 04510, Mexico; fmendez@unam.mx

**Keywords:** steric effect, power-law fluids, boundary slip, oscillatory electroosmotic flow, mass transport rate

## Abstract

In this paper, the combined effect of the fluid rheology, finite-sized ions, and slippage toward augmenting a non-reacting solute’s mass transport due to an oscillatory electroosmotic flow (OEOF) is determined. Bikerman’s model is used to include the finite-sized ions (steric effects) in the original Poisson-Boltzmann (PB) equation. The volume fraction of ions quantifies the steric effects in the modified Poisson-Boltzmann (MPB) equation to predict the electrical potential and the ion concentration close to the charged microchannel walls. The hydrodynamics is affected by slippage, in which the slip length was used as an index for wall hydrophobicity. A conventional finite difference scheme was used to solve the momentum and species transport equations in the lubrication limit together with the MPB equation. The results suggest that the combined slippage and steric effects promote the best conditions to enhance the mass transport of species in about 90% compared with no steric effect with proper choices of the Debye length, Navier length, steric factor, Womersley number, and the tidal displacement.

## 1. Introduction

Lab-on-a-chip technology requires the manipulation and control of fluid flow to transport, mixing, and separation of reagents in nanoliter volumes in microfluidic devices widely used in chemical, medical, and biological applications, among others. These tasks are typically difficult to achieve because the laminar viscous flow governs electrokinetic transport phenomena (electroosmosis and electrophoresis) and due to the small mass-diffusivities of the species. In these applications, a broad kind of fluids are handled inside the microfluidic devices, from simple electrolytic solutions treated as Newtonian fluids to complex cell suspensions, biological fluids, such as blood, saliva, and DNA solutions, and polymer melts where viscosity is assumed to depend on the shear rate (i.e., the fluid is non-Newtonian). Therefore, understanding the fundamental behavior of the combined effects of fluid rheology, interfacial phenomena (steric and slippage effects), and the flow behavior in mass transport of a neutral solute through pure electroosmotic flow (EOF) or oscillatory electroosmotic flow (OEOF) is essential for the analysis and design of microfluidic components, such as microchannels, micro-mixers, and micro-pumps, that can be implemented in the design of biochips.

Taylor [1] was the first to establish that dispersion of a soluble substance (solute) driven by a Poiseuille flow in a circular cylindrical tube is controlled by the coefficient of diffusivity, which can be calculated directly from the solute profile. Aris [2] reported alternative treatments to Taylor’s analysis of simultaneous convection and diffusion in dispersion. Likewise, when a solute is introduced into a pulsating flow through a circular tube, the solute has an increased mass transfer beyond molecular diffusion due to Taylor dispersion. The transport and separation phenomena of mass species are critical steps in realizing lab-on-a-chip. These steps are complicated because microfluidic devices manipulate flows with Reynolds numbers small enough for inertial effects to be irrelevant and species with small mass-diffusivity coefficients with magnitude $D∼O(10^{-9})$ m^2^ s^−1^ [3]. In the case of a pure EOF, where a typical plug-like velocity profile exists, it does not influence species’ transport and dispersion processes, and the mass transport is governed by pure diffusion.

Oscillatory and pulsating flows have mainly focused on enhancing the dispersion or separation of a passive solute under different conditions [4,5,6]. Kurzweg and Jaeger [7] performed the separation process with oscillatory flows of different species in which the lower diffuser, under a specific frequency, travels faster than the rapid diffuser to achieve the cross-over condition. Likewise, Thomas and Narayanan [8] showed how particles subjected to oscillatory and pulsating motions have a zigzag movement during mass transport, and that is why the transport of species is improved.

The dispersion of a neutral solute in an EOF was improved by oscillatory effects induced in the flow, i.e., the mass transport is caused by an oscillatory electroosmotic flow (OEOF) [9,10,11,12]. Despite the low diffusivity of the species in liquid solutions, time-periodic electroosmotic flow (AC) has a significant advantage over pure electroosmotic flows (DC) in biotechnology and physical separation methods. Hence, in this context, OEOF is a good candidate as a viable mechanism to induce efficient separation processes [13] due to the cross-over phenomenon between two solutes with two different mass-diffusivity coefficients [14].

The steric effect is an interfacial phenomenon in electrokinetics that frequently occurs in microfluidic confinements. EOF and OEOF are founded in electroosmosis, which refers to ionized liquid’s motion relative to the stationary charged surface by an applied external electric field. The ionic size effects are controlled by the mean volume fraction of each ion in bulk given by $\nu =a^{3}n_{0}$ [15], where *a* is the effective ion size, and $n_{0}$ is the bulk number concentration. Recently, studies have analyzed the steric effects using Bikerman’s modified Poisson-Boltzmann (MPB) equation to account for the crowding of finite-sized ions in EOF by considering: non-linear biofluids, such as solutions of blood, saliva, protein, DNA, polymeric solutions, and colloidal suspensions; these fluids reveal non-linear rheology encountered in biomicrofluidic systems using the power-law viscosity model [16]; step-change in the wall temperature [17]; viscoelectric effects [18]; wall slip effects and steric interactions [19]; and using OEOF few works have been conducted [20]. The vast majority of theoretical work on colloidal electrokinetics using OEOF utilizes the Poisson–Boltzmann equation, wherein ions are treated as point charges and non-interacting. The slip condition is another interfacial phenomenon that enhances the electrokinetic effects (electrophoresis, electro-osmosis, streaming current or potential, etc.) [21]. In the literature concerning mass transport under slippage effect, Munoz et al. [22] considered oscillatory electroosmotic flow, and they found that the dispersivity may be maximized up to two orders of magnitude compared with that obtained using the classical no-slip condition. Moreover, the mass transport and separation of species in OEOF can be improved by controlling the external electrical signal type [23].

The dispersion of solutes in physiological systems, where fluids are significantly more viscous, the effects of non-Newtonian rheologies, such as shear thinning, could also be considered, due to that the mass transport of neutral species is the result of an interaction between transverse diffusion and the structure of the flow. Hydrodynamics and dispersion phenomena of EOF were addressed in the context of non-Newtonian fluids [24,25,26]. For instance, recently, the unsteady solute dispersion by electrokinetic flow was studied [27] by considering wall absorption. Besides, some studies were conducted on the hydrodynamics of the OEOF for non-Newtonian fluids [28,29,30], and, recently, the mass transfer of an electroneutral solute in a concentric-annulus microchannel driven by an OEOF for a fluid whose behavior follows the Maxwell model was reported by Peralta et al. [31]. However, minimal effort has been devoted to understanding the fundamental physics that can serve us to improve mass transport and species separation under the influence of combined effects of rheology, finite ion size, and slippage that are present in the microfluidic devices.

In previous works, the analysis of dispersion and separation of neutral solutes has been performed on OEOF using non-Newtonian fluids. In contrast, this paper analyzes the combined effects of finite-sized ions, fluid rheology, and slippage on the mass transport of a neutral solute; these effects are controlled by the steric factor ν, the power law-index *n*, and the Navier slip length $\lambda_{N}$, respectively. A common non-linear rheological model for a fluid with shear-dependent viscosity is the power-law model since it efficiently describes, for engineering and microfluidics purposes, the rheology of many fluid substances over a wide range of shear rates. Although the main deficiency of the power-law model is the divergence of the effective viscosity in the limits of both zero and very large shear rates, it has the advantage of both applicability and simplicity to justify its use in investigations of shear-dependent flow behaviors. In general, lab-on-a-chip devices are designed to carry out the following functions: sample introduction, injection, mixing, reaction, transport, separation, and detection through a series of micrometre-scale channels [32]. In this context, to understand the fundamental physics, as well as provide useful information and criteria for designing micro-fluidic devices, the present study is, thus, aimed at the theoretical investigation of the transport and separation phenomena of mass species in an OEOF in a microchannel. Additionally, the start-up from the rest of the flow was analyzed, and, for larger times after the initial transient has died out, the flow was periodic in time, and then the concentration and mass transport rate were determined.

## 2. Problem Formulation

**Figure 1:**
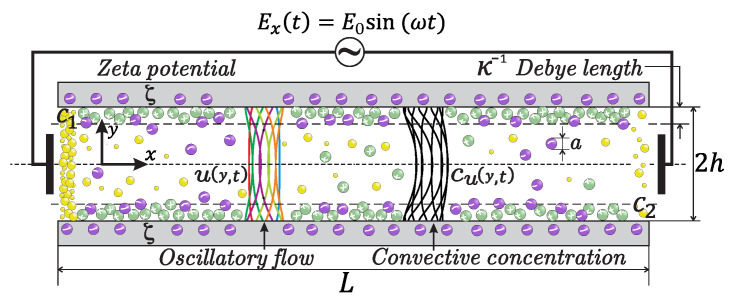
Schematic depiction for mass transport due to an oscillatory electroosmotic flow.

The sketch in Figure 1 represents the physical model of the OEOF through a long horizontal microchannel. It consists of two parallel plates separated by a distance (height) of 2h. The length of the microchannel is *L*, and the depth in the *z*-direction is *W*, both assumed to be much larger than the height, i.e., L,W>>2h; therefore, the flow field is assumed independent of the *z* coordinate. The origin of the cartesian coordinate system (*x*, *y*) is located at the left end and at the center of the microchannel. The microchannel is filled with a non-Newtonian liquid that obeys the power-law rheological model, which connects two reservoirs at the ends, such that the concentration c(x,y,t) of the non-reacting solute at the left-reservoir is maintained at a constant concentration $C_{1}$, while the other extreme is found to a prescribed uniform concentration $C_{2}$ and assuming that $C_{1}>C_{2}$. Besides, the effect of a certain degree of slip at the microchannel walls, quantified by the slip length $\lambda_{N}$, is also considered [33]. The OEOF will occur in the microchannel under the simultaneous influence of the externally imposed oscillatory electric field $E_{x}\left(t\right)$ and the induced electric field into the non-overlapping EDLs. The microchannel walls are charged with a uniform high zeta potential ζ enough to cause crowding of ions of the dilute solution near the surface walls [15]. In this context, the zeta potential is several times higher than the thermal voltage $k_{B}T/ze$ in the MPB with ν≠0 (representing ionic size effects), and $z,e,k_{B}$, and *T* are the valency of ions, the magnitude of the fundamental charge on an electron, the Boltzmann constant and the absolute temperature, respectively. The start-up of this OEOF from rest occurs, and, for sufficiently long times after imposition of $E_{x}\left(t\right)$, the velocity field is strictly periodic.

### 2.1. Electrical Field: Steric Effects

When an electrolyte solution is in contact with a uniformly charged surface, electrical (zeψ) and chemical ($k_{B}T\ln n_{\pm }$) effects modify the electro-chemical potential $\mu_{\pm }$ (±denotes the sign of the charge ze); then, the electrochemical potential derives in $\mu_{\pm }=\pm ze\psi -k_{B}T\ln n_{\pm }$, where ψ is the electric potential, and $n_{\pm }$ is the ionic concentration in the diffuse electric double layer. Positive and negative ions are separated within the diffuse double layer by Boltzmann distribution $n_{\pm }=n_{0}e^{\mp ze\psi /k_{B}T}$, where an equilibrium exists between external electric and osmotic forces. The electrical potential ψ is determined with the classical complete Poisson-Boltzmann equation $\epsilon \nabla^{2}\Phi =-\rho_{e}$. Here, Φ=ϕ(x,t)+ψ(y) is defined by the linear superposition [34] of the local electric potential ϕ(x,t) and the corresponding potential ψ(y) induced into the EDL, ϵ is the permittivity of the solution. The volume charge density $\rho_{e}$ in the neighborhood of the surface is $\rho_{e}=ze\left(n_{+}-n_{-}\right)$. However, the above treatment is valid if ions are treated as electric charges having no volume [35] and no other individual effects [36]. The present investigation involves large potentials; then the Poisson-Boltzmann equation has shortcomings because it neglects steric effects. The modified PB equations that consider the steric effects in $n_{\pm }$ due to finite-sized ions [15] is derived from the modified chemical potential $\mu_{\pm }$ using Bikerman’s model [37], given by

(1) $$\mu_{\pm }=\pm ze\psi -k_{B}T\ln n_{\pm }-k_{B}T\ln\left(1-n_{+}a^{3}-n_{-}a^{3}\right).$$

The third term on the right-hand side of Equation (1) considers the finite-sized ions. Under equilibrium conditions, the ions’ electrochemical potential is constant $\nabla \mu_{\pm }$ = 0, and it derives in:

(2) $$\frac{\nabla n^{\pm }}{n^{\pm }}-\frac{\nabla (1-n_{+}a^{3}-n_{-}a^{3})}{1-n_{+}a^{3}-n_{-}a^{3}}=\mp \frac{ze}{k_{B}T}\nabla \psi .$$

Integrating Equation (2) from a point in the bulk solution (where ψ=0 and $n_{\pm }=n_{\infty }$) leads to the modified Boltzmann distribution:

(3) $$n_{\pm }=\frac{n_{0}\exp\left(\pm ze\psi /k_{B}T\right)}{1+2\nu \sinh^{2}\left(ze\psi /2k_{B}T\right)}.$$

Substituting ion distribution $n_{\pm }$ into the volume charge density $\rho_{e}$ takes the following form:

(4) $$\rho_{e}=-2zen_{0}\frac{\sinh(ze\psi /k_{B}T)}{1+2\nu \sinh^{2}\left(ze\psi /2k_{B}T\right)},$$

where $\nu =2a^{3}n_{0}$ is the bulk volume fraction of ions. To account for steric effects associated with the finite-sized ions and solvent molecules, Equation (3) was combined with the complete Poisson equation yielding the MPB equation:

(5) $$\nabla^{2}\Phi =\frac{2zen_{0}}{\epsilon }\frac{\sinh(ze\psi /k_{B}T)}{1+2\nu \sinh^{2}\left(ze\psi /2k_{B}T\right)}.$$

Equation (5) is essentially a modified form of the PB equation considering finite-sized ions effects. The omission of the temporal term in Poisson’s equation is because the characteristic time scale (∼$10^{-12}$ s) of the electro-migration in the EDL is much less than the corresponding time-scale (∼$10^{-2}$ s) for the viscous diffusion [38].

For a long microchannel, L≫h, the term $\partial^{2}\Phi /\partial x^{2}$ in Equation (5) may be neglected [39]. One gets the simplified version of the MPB equation given by

(6) $$\frac{\;d^{2}\psi }{\;dy^{2}}=\frac{2zen_{0}}{\epsilon }\frac{\sinh(ze\psi /k_{B}T)}{1+2\nu \sinh^{2}\left(ze\psi /2k_{B}T\right)}.$$

The boundary conditions of Equation (6) are dψ/dy=0 at y=0 and ψ=ζ at y=h. Using the following dimensionless variables $\bar{y}=y/h$ and $\bar{\psi }=ze\psi /k_{B}T$ into Equation (6), it derives in:

(7) $$\frac{\;d^{2}\bar{\psi }}{\;d{\bar{y}}^{2}}={\bar{\kappa }}^{2}\frac{\sinh\bar{\psi }}{1+2\nu \sinh^{2}\left(\bar{\psi }/2\right)},$$

with the corresponding boundary conditions,

(8) $$\bar{\psi }=\kappa_{\psi }\;\mathrm{at}\;\bar{y}=1,$$

and

(9) $$\frac{d\bar{\psi }}{d\bar{y}}=0\;\mathrm{at}\;\bar{y}=0.$$

The parameter $\bar{\kappa }=\kappa h$ is the ratio of the microchannel height to the Debye length, defined by $\kappa^{-1}=\epsilon k_{B}T/2e^{2}z^{2}n_{0}$. The competition between the wall ζ potential and the thermal potential is given by $\kappa_{\psi }=ze\zeta /k_{B}T$. When $\kappa_{\psi }<1$, it means low ζ potentials. In contrast, for $\kappa_{\psi }>>1$, it represents the case of high ζ potentials. In this work, $\kappa_{\psi }=2$, and high zeta potentials $\kappa_{\psi }=10$ were considered since, in dilute liquids, the steric effects are visible at high zeta potentials at which high ionic concentrations at the microchannel wall cause extreme accumulation of counterions [40].

### 2.2. Velocity Field

By assuming that the microchannel is very long and focusing on the central region, away from the microchannel entry and exit, it is assumed that the flow is unidirectional and fully developed. Therefore, the momentum equation is given by

(10) $$\rho_{f}\frac{\partial u}{\partial t}=\frac{\;\partial \tau_{xy}}{\;\partial y}+\rho_{e}E_{x}\left(t\right).$$

Here, u(y,t) is the velocity component in the *x* direction, $\rho_{f}$ is the mass density and $\tau_{xy}$ represents the shear stress. Equation (10) is subject to the symmetry boundary condition of the velocity (∂u/∂y=0) at the center of the microchannel. The Navier slip boundary condition at the interface between the fluid and the microchannel wall is considered, given by $u_{s}=\lambda_{N}\left\{D\cdot n-\left[\left(D\cdot n\right)\cdot n\right]n\right\}$[41]. Here, $u_{s}$ is the fluid velocity at the microchannel wall, n represents the unit vector normal to the microchannel surface pointing toward the fluid, the rate of strain tensor is defined as $D=\nabla u+{\left(\nabla u\right)}^{tr}$, u is the velocity field, and tr denotes the transpose of ∇u. For the present problem, the slip boundary condition is simplified to

(11) $$u=-\lambda_{N}\frac{\partial u}{\partial y}\;\mathrm{at}\;y=h.$$

Besides, it is assumed that the fluid is at rest for t=0, that is,

(12) u=0att=0,for−h⩽y⩽h.

The shear stress $\tau_{xy}$ for non-Newtonian fluids where the dynamic viscosity $\eta (\dot{\gamma })$ is a function of the strain rate $\dot{\gamma }$, is defined as $\tau_{xy}=\eta \left(\dot{\gamma }\right)\dot{\gamma }$. For the unidirectional and fully developed OEOF, $\dot{\gamma }=\partial u/\partial y$, and the dynamic viscosity $\eta (\dot{\gamma })$ is a function of the velocity gradient, according to the power-law model as follows [42]:

(13) $$\eta \left(\dot{\gamma }\right)=m{\left(\frac{\partial u}{\partial y}\right)}^{\left(n-1\right)},$$

where *m* denotes the consistency index, and *n* is the power-law index. Thus, substituting $\rho_{e}$ defined in Equation (4) and $E_{x}\left(t\right)$ into (10), it derives in

(14) $$\rho_{f}\frac{\partial u}{\partial t}=\frac{\partial }{\partial y}\left\{\eta \frac{\partial u}{\partial y}\right\}-\frac{2zen_{\infty }\sinh\left(ze\psi /k_{B}T\right)}{1+2\nu \sinh^{2}\left(ze\psi /2k_{B}T\right)}E_{0}\sin\left(\omega t\right).$$

Using the following dimensionless variables, $\bar{u}=u/U_{HS}$, $\bar{y}=y/h$, τ=ωt/2π, where $U_{HS}=-\epsilon \zeta_{0}E_{0}/\mu$ is the Helmholtz-Smoluchowski velocity. Therefore, the dimensionless version of the momentum Equation (14) is as follows:

(15) $$\begin{array}{c} \frac{Wo^{2}}{2\pi }\frac{\partial \bar{u}}{\partial \tau }=\bar{m}\left[\bar{\eta }\frac{\partial^{2}\bar{u}}{\partial {\bar{y}}^{2}}\right]-\frac{{\bar{\kappa }}^{2}}{\kappa_{\psi }}\frac{\sinh\bar{\psi }}{1+2\nu \sinh^{2}\left(\bar{\psi }/2\right)}\sin\left(2\pi \tau \right), \end{array}$$

where the dimensionless dynamic viscosity is defined as

(16) $$\bar{\eta }={\left(\frac{\partial \bar{u}}{\partial \bar{y}}\right)}^{\left(n-1\right)}.$$

The parameter $\bar{m}=n\left(m/\mu \right){\left(U_{HS}/h\right)}^{n-1}$ is the dimensionless consistency index and relates the characteristic shear stresses for Non-Newtonian and Newtonian fluids. The Womersley number is defined as $Wo=h\sqrt{\omega /\nu_{0}}$ and relates the ratio of the viscous diffusion time-scale to the oscillation time-scale, where $\nu_{0}=\mu /\rho_{f}$. Here, it is important to note that the Womersley is based on physical properties of a Newtonian fluid. This is because in the process of nondimensionalizing the mathematical problem, the characteristic scale for the velocity, $U_{HS}$, corresponds to the classical Helmholtz-Smoluchowsky velocity [39], where the viscosity μ of a Newtonian fluid is considered. However, the non-Newtonian parameters of the Power-Law fluid are taken into account in the definitions of the dimensionless parameter $\bar{m}$. Equation (15) is subject to the following dimensionless boundary and initial conditions:

(17a) $$\begin{array}{c} \frac{\partial \bar{u}}{\partial \bar{y}}=0\;\mathrm{at}\;\bar{y}=0, \end{array}$$

(17b) $$\begin{array}{c} \bar{u}=-\delta \frac{\partial \bar{u}}{\partial \bar{y}}\;\mathrm{at}\;\bar{y}=1, \end{array}$$

(17c) $$\begin{array}{c} \bar{u}=0\;\mathrm{at}\;\tau =0\;\mathrm{for}\;-1⩽\bar{y}⩽1. \end{array}$$

Here, $\delta =\lambda_{N}/h$ denotes the ratio between the Navier length and the microchannel height.

### 2.3. Concentration Field

For the fully developed OEOF described in Section 2.2, diffusion and convection mechanisms govern the mass transport of passive species. Assuming that the transport phenomenon is not affected by any of the electrical potentials, and the particles do not interact with each other, the concentration field of the solute c(x,y,t) with constant molecular diffusion coefficient *D* can be found by solving the species conservation equation, given by [39]

(18) $$\frac{\partial c}{\partial t}+u\frac{\partial c}{\partial x}=D\left(\frac{\partial^{2}c}{\partial x^{2}}+\frac{\partial^{2}c}{\partial y^{2}}\right).$$

The boundary and initial conditions associated to Equation (18) are

(19a) $$\begin{array}{c} c\left(y,t\right)=C_{1}\;\mathrm{at}\;x=0, \end{array}$$

(19b) $$\begin{array}{c} c\left(y,t\right)=C_{2}\;\mathrm{at}\;x=L, \end{array}$$

(19c) $$\begin{array}{c} \frac{\partial c(x,t)}{\partial y}=0\;\mathrm{at}\;y=h, \end{array}$$

(19d) $$\begin{array}{c} \frac{\partial c(x,t)}{\partial y}=0\;\mathrm{at}\;y=0, \end{array}$$

(19e) $$\begin{array}{c} c\left(x\right)=C_{1}+\left(C_{2}-C_{1}\right)\left(x/L\right)\;\mathrm{at}\;t=0. \end{array}$$

In Equations (19a) and (19b), $C_{1}$ and $C_{2}$ are fixed values of c(y,t) at the left and right reservoirs, respectively. As depicted in Figure 1, $C_{1}>C_{2}$. Equations (19c) and (19d) denote the impermeability and symmetry conditions, respectively. Here, a linear profile of the concentration distribution is imposed at t=0, prescribed in Equation (19e). Because of the linearity of Equation (18), it is often convenient to use the Chatwin approximation [4,43], given by the superposition of a linear distribution for the concentration and other corresponding with convective effects by the oscillatory motion of the fluid, which is given by

(20) $$c\left(x,y,t\right)=C_{1}+\frac{C_{2}-C_{1}}{L}x+c_{u}\left(y,t\right).$$

This approximate solution is invalid near the microchannel ends due to that Equation (20) does not satisfy the boundary conditions at both ends taking into account that $c_{u}\left(y,t\right)$ is different to zero. However, it is valid for long microchannels (L≫h) where any end effects are neglected [13,43]. Substituting Equation (20) into Equation (18) yields

(21) $$\frac{\partial c_{u}}{\partial t}+u\left(\frac{C_{2}-C_{1}}{L}\right)=D\frac{\partial^{2}c_{u}}{\partial y^{2}}$$

with the following boundary and initial conditions,

(22a) $$\begin{array}{c} \frac{\partial c_{u}\left(t\right)}{\partial y}=0\;\mathrm{at}\;y=h, \end{array}$$

(22b) $$\begin{array}{c} \frac{\partial c_{u}\left(t\right)}{\partial y}=0\;\mathrm{at}\;y=0, \end{array}$$

(22c) $$\begin{array}{c} c_{u}\left(y\right)=0\;\mathrm{at}\;t=t_{\omega }. \end{array}$$

In Equation (22c), $t_{\omega }$ refers to the value of *t* when the initial transient step has died out. Introducing the dimensionless concentration of species as ${\bar{c}}_{u}=c_{u}/\left(C_{2}-C_{1}\right)$ and the dimensionless variables defined before, Equation (21) can be rewritten in the following form:

(23) $$\frac{Wo^{2}Sc}{2\pi }\frac{\partial {\bar{c}}_{u}}{\partial \tau }+Pe_{D}\alpha \bar{u}=\frac{\partial^{2}{\bar{c}}_{u}}{\partial {\bar{y}}^{2}},$$

where $Sc=\nu_{0}/D$ is the Schmidt number that measures the competition between the momentum and the mass diffusivities. $Pe_{D}=U_{HS}h/D$ is the diffusive Péclet number, measuring the ratio of the convective to the diffusive transport rate. Here, in a similar manner as the definition of the Womersley number, the Schmidt and Péclet numbers are based on physical properties of a Newtonian fluid. However, in the convection-diffusion Equation (23), the influence of the fluid’s reology is taken into account in the velocity profile $\bar{u}\left(\bar{y},\bar{\tau }\right)$. Hence, the dimensionless form of the boundary and initial conditions (22a)–(22c) are:

(24a) $$\begin{array}{c} \frac{\partial {\bar{c}}_{u}}{\partial \bar{y}}{|}_{\bar{y}=1}=0, \end{array}$$

(24b) $$\begin{array}{c} \frac{\partial {\bar{c}}_{u}}{\partial \bar{y}}{|}_{\bar{y}=0}=0, \end{array}$$

(24c) $$\begin{array}{c} {\bar{c}}_{u}\left(\bar{y},\tau =\tau_{\omega }\right)=0. \end{array}$$

In Equation (24c), $\tau_{\omega }$ denotes the value of the dimensionless time τ from which the flow becomes periodic, i.e., the transient stage has died out. In physical units, such condition is achieved when the time *t* assumes a value of the characteristic scale of the viscous diffusion time-scale ($t∼h^{2}/\nu_{0}$). On the other hand, for the periodic condition of the flow, $1/\omega ∼h^{2}/\upsilon$, and, taking into account the definition of the dimensionless time, it yields that $\tau_{\omega }=t_{\omega }\omega /2\pi ∼O\left(10^{1}\right)$. This value of $\tau_{\omega }$ is used for all the numerical calculations.

### 2.4. Mass Transport Rate

The time-averaged mass transfer in the system $Q_{x}$ was evaluated during one period of oscillation, defined by [43]:

(25) $$Q_{x}=\frac{1}{4h}\frac{\omega }{\pi }\int_{-h}^{h}\int_{t_{\omega }}^{t_{\omega }+\frac{2\pi }{\omega }}J_{x}dtdy,$$

where $J_{x}$ represents the total flux density defined as the sum of convective $j_{x,conv}$ and diffusive $j_{x,diff}$ flux densities in the *x*-direction, as follows:

(26) $$J_{x}=j_{x,conv}+j_{x,diff}=u\left(y,t\right)c\left(x,y,t\right)-D\frac{\partial c(x,y,t)}{\partial x}.$$

Substituting Equation (26) into Equation (25), the dimensionless rate of mass transport ${\bar{Q}}_{x}$ in terms of dimensionless variables is as follows

(27) $${\bar{Q}}_{x}=1-\frac{Pe_{D}}{2\alpha }\int_{-1}^{1}\int_{\tau_{\omega }}^{\tau_{\omega }+1}\bar{u}{\bar{c}}_{u}d\bar{y}d\tau .$$

Here, ${\bar{Q}}_{x}=Q_{x}L/\left(C_{1}-C_{2}\right)D$. Due to the flow’s oscillatory character, the Helmholtz-Smoluchwosky velocity $U_{HS}$, and the corresponding Péclet number, $Pe_{D}$, will no longer remain constant for particular values of the angular frequency ω, or consequently Wo. To show the frequency dependence of ${\bar{Q}}_{x}$, it is necessary to use the concept of tidal displacement Δz. This quantity is defined as the cross-stream averaged maximum axial distance for which the fluid elements travel during the one-half period of the oscillation [13],

(28) $$\Delta z=\frac{1}{2h\pi }|\int_{-h}^{h}\int_{t_{\omega }}^{t_{\omega }+\frac{\pi }{\omega }}u\;dt\;dy|.$$

After introducing the dimensionless variables, $\bar{u}=u/U_{HS}$, $\bar{y}=y/h$, and τ=ωt/2π into Equation (29), can be simplified in the following form:

(29) $$\Delta z=\frac{U_{HS}}{\omega }|\int_{-1}^{1}\int_{\tau_{\omega }}^{\tau_{\omega }+1/2}\bar{u}\;d\tau \;d\bar{y}|.$$

From Equation (29), the Helmholtz-Smoluchwosky velocity $U_{HS}$ is obtained as:

(30) $$U_{HS}=\frac{\omega \Delta z}{\left|\int_{-1}^{1}\int_{\tau_{\omega }}^{\tau_{\omega }+1/2}\bar{u}\;d\tau \;d\bar{y}\right|},$$

where it is evident the frequency dependence of $U_{HS}$. Substituting Equation (30) into the Péclet number definition, it provides a relationship as a function of Sc, Wo, and ΔZ, as follows:

(31) $$Pe_{D}=\frac{Wo^{2}Sc\Delta Z}{\left|\int_{-1}^{1}\int_{\tau_{\omega }}^{\tau_{\omega }+1/2}\bar{u}\;d\tau \;d\bar{y}\right|}.$$

For different fixed values of the dimensionless tidal displacement ΔZ=Δz/h, significant changes in $Pe_{D}$ are obtained for each flow situation. In this context, the concentration field and the overall mass transfer will be affected by the frequency.

## 3. Numerical Scheme

**Figure 2:**
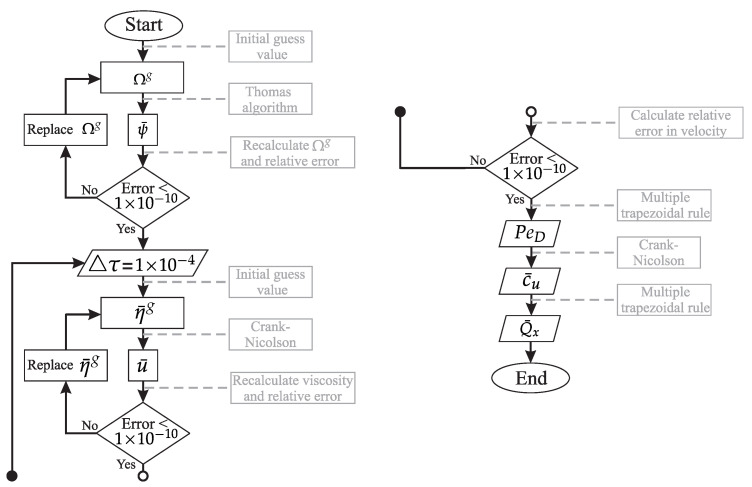
Schematic diagram of the numerical algorithm.

Figure 2 shows a flowchart of the methodology used to solve the formulated problem. The algorithm was developed in Fortran PowerStation version 4.0 with Microsoft Developer Studio software; the process is described more precisely as indicated below.

### 3.1. Electric Potential Field

The MPB equation (7) was approximated by the second-order centered-space difference, it derives in

(32) $${\bar{\psi }}_{i+1}-\left(2+\Delta {\bar{y}}^{2}{\bar{\kappa }}^{2}\Omega^{g}\right){\bar{\psi }}_{i}+{\bar{\psi }}_{i-1}=0,$$

where

(33) $$\Omega^{g}=\sinh\left({\bar{\psi }}_{i}\right)/{\bar{\psi }}_{i}\left[1+2\nu \sinh^{2}\left({\bar{\psi }}_{i}/2\right)\right].$$

For an initial guess value in $\Omega^{g}$, the equation system in (32) can be solved simultaneously by applying the Thomas algorithm [44]. Using the solution obtained for $\psi_{i}$, the value of Ω is recalculated according to (33), and the new value replaces the previous one. This process is repeated until a numerical error value of 1 × $10^{-10}$ is reached.

### 3.2. Velocity Field

Equation (15) was solved using the Crank-Nicolson method, applying a central difference scheme [44]. The resulting discretization of Equation (15) is as follows:

(34) $$-\theta_{1}{\bar{u}}_{i+1}^{l+1}+\theta_{2}{\bar{u}}_{i}^{l+1}-\theta_{1}{\bar{u}}_{i-1}^{l+1}=\theta_{3},$$

where $\theta_{1}$, $\theta_{2}$, and $\theta_{3}$ are defined as:

(35a) $$\begin{array}{c} \theta_{1}=\frac{{\bar{\eta }}^{g}\gamma }{2\Delta {\bar{y}}^{2}}, \end{array}$$

(35b) $$\begin{array}{c} \theta_{2}=2\theta_{1}+\frac{1}{\Delta \tau }, \end{array}$$

(35c) $$\begin{array}{c} \theta_{3}=\frac{{\bar{u}}_{i}^{l}}{\Delta \tau }+\theta_{1}\left({\bar{u}}_{i+1}^{l}-2{\bar{u}}_{i}^{l}+{\bar{u}}_{i-1}^{l}\right)+\Lambda , \end{array}$$

and

(36) $${\bar{\eta }}^{g}={\left[{\left(\frac{{\bar{u}}_{i+1}^{l}-{\bar{u}}_{i}^{l}}{\Delta \bar{y}}\right)}^{2}\right]}^{\left(\frac{n-1}{2}\right)}.$$

Here, Δτ and $\Delta \bar{y}$ are the time step and the size step in the $\bar{y}$ direction, respectively. In this work, a $\Delta \bar{y}$-step of 2 × $10^{-3}$ and Δτ-step of 1 × $10^{-4}$ have been used in all numerical essays. The parameters γ and Λ in Equations (35a) and (35c) are defined as follows:

(37) $$\gamma =\frac{2\pi \bar{m}}{Wo^{2}},$$

and

(38) $$\Lambda =\frac{2\pi {\bar{\kappa }}^{2}}{Wo^{2}\kappa_{\psi }}\frac{\sinh{\bar{\psi }}_{i}}{1+2\nu \sinh^{2}\left({\bar{\psi }}_{i}/2\right)}\sin\left(2\pi \tau^{l}\right).$$

The numerical value of $\psi_{i}$ is provided in (38) by the iterative procedure described in (32) and (33). To solve the velocity field a similar procedure to that explained in the previous section was applied. The solution begins by providing an initial guess value to ${\bar{\eta }}^{g}$. To solve the non-linear equation system generated by (34), a tridiagonal matrix algorithm (TDMA) was used. With the values obtained for the velocity field ${\bar{u}}_{i+1}^{l+1}$, the term ${\bar{\eta }}^{g}$ is recalculated at the next iteration and the process is repeated until the required relative error is achieved. Given that Equation (36) is a function of $\bar{y}$, it was recalculated for each node in the space and time. This solution procedure is useful because, when $\partial \bar{u}/\partial \bar{y}=0$ with an index *n* smaller than unity, the value of ${\bar{\eta }}^{g}$ is undetermined; thereby, a numerical value very close to zero is assigned to ${\bar{\eta }}^{g}$ when $\partial \bar{u}/\partial \bar{y}\to 0$ to avoid a singularity.

### 3.3. Concentration Field and Mass Transport Rate

After solving the electrical potential and the velocity field, the Péclet number has to be determined. Average velocity is required as it is indicated in Equation (31), this average was calculated using the multiple-trapezoid rule. The concentration field in Equation (23) was approximated by using the second-order central-difference formula for the second derivative diffusion term and the forward difference formula for the first-order time derivative. Then, the concentration equation can be discretized as follows:

(39) $$-\beta_{2}{\bar{c}}_{u,i+1}^{n+1}+\left(1+2\beta_{2}\right){\bar{c}}_{u,i}^{n+1}-\beta_{2}{\bar{c}}_{u,i-1}^{n+1}=\beta_{1},$$

where

(40a) $$\begin{array}{c} \beta_{1}=-2\beta_{2}\Delta {\bar{y}}^{2}Pe_{\omega }\alpha {\bar{u}}_{i}^{n}+{\bar{c}}_{u,i}^{n}+\beta_{2}\left({\bar{c}}_{u,i+1}^{n}-2{\bar{c}}_{u,i}^{n}+{\bar{c}}_{u,i-1}^{n}\right), \end{array}$$

(40b) $$\begin{array}{c} \beta_{2}=\frac{\pi \Delta \tau }{Wo^{2}Sc\Delta {\bar{y}}^{2}}. \end{array}$$

Finally, with the results obtained for $\bar{u}$ and ${\bar{c}}_{u}$, the dimensionless rate of mass transport ${\bar{Q}}_{x}$ defined in Equation (27) is calculated by applying the multiple-trapezoidal rule. The dimensionless initial condition ${\bar{T}}_{\omega }$ for the concentration field can be any value of the non-dimensional time in such a way that the transient stage for the velocity field has diet out.

A description of the validation process between the numerical solution and the analytical solution of the velocity field reported by Huang and Lai [13] is presented. The main differences between both studies are the following: The flow configuration considered by Huang and Lai [13] is a two-dimensional rectangular microchannel of length *L* and width *h* filled with a Newtonian liquid which is an electrolyte. The coordinate system (x,y) is located on the lower wall at the microchannel inlet. The associated boundary conditions for the velocity are the no-slip condition at the channel walls.

**Figure 3:**
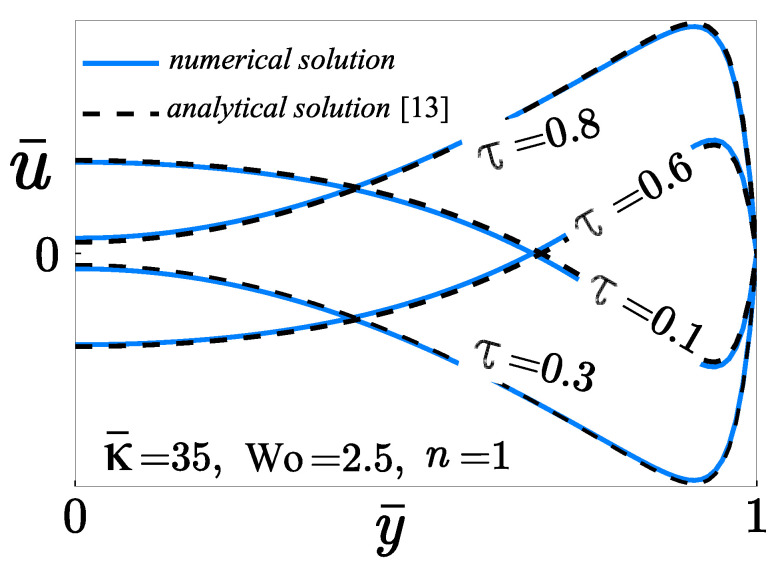
Comparison of the analytical solution [13] and the numerical solution (present work) for the dimensionless velocity $\bar{u}$ across the microchannel in a Newtonian fluid.

Conversely, in the present study, the microchannel consists of two parallel plates with a length *L* and distant by 2h. The origin of the cartesian coordinate system (x,y) is located to the left end of the microchannel center. A non-Newtonian liquid that obeys the power-law rheological model flows through the microchannel. For the hydrodynamics, the problem formulation includes the effects of slip, which enter the problem through a Navier-slip model, and the symmetry boundary condition at the microchannel’s midplane. The dimensionless velocity distribution across the channel-width with a Womersley number W=5 and a dimensionless Debye length $\lambda^{*}=70$ reported by Huang and Lai corresponds to the velocity profile with Wo=2.5 and $\bar{\kappa }=35$ in the present study assuming the slippage is absent, δ=0, with a power-law index n=1. An excellent agreement between both solutions is observed in Figure 3.

## 4. Results and Discussion

This section highlights the numerical solution of the MPB, momentum, and conservation species equations, and mass transport rate provided in a microchannel due to an OEOF of power law fluids by considering the steric and slippage effects. For pertinent results, the appropriate dimensionless parameters are highlighted in Table 1, by considering the relevant geometrical and physicochemical properties, as shown in Table 2.

**Figure 4:**
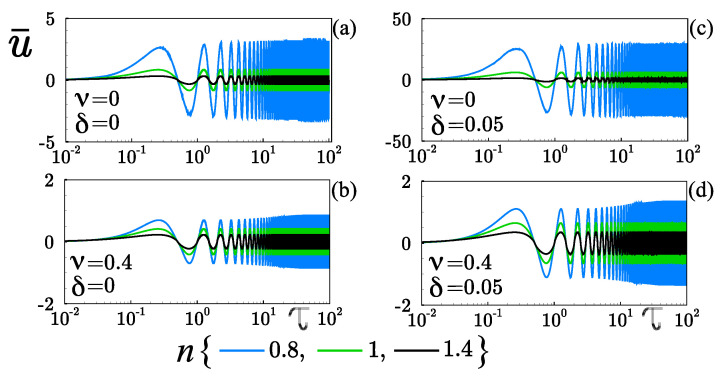
Evolution over time of the dimensionless velocity $\tilde{u}$ at $\bar{y}=0.9$, affected by the slippage (δ) and the finite-sized ions (ν). Here, Wo=0.5, $\bar{\kappa }=20$, and $\kappa_{\psi }=10$. (**a**) ν=0, δ=0; (**b**) ν=0.4, δ=0; (**c**) ν=0, δ=0.05 and (**d**) ν=0.4, δ=0.05.

In Figure 4, the absence and the presence of the steric effect and the slippage or the combined effects on the evolution over time of the velocity $\bar{u}$ for Newtonian and non-Newtonian fluids is presented. The blue, black, and green lines represent the flow solution for Shear-thinning (with n=0.8), Shear-thickening (with n=1.4), and Newtonian (with n=1) behavior of the fluid, respectively. Once the electric field $E_{x}\left(t\right)$ is applied, the flow initiates the transient state where the oscillatory effects have no relevance; that is, the velocity exhibits a gradual increase in its magnitude, and the periodic flow behavior starts when τ∼O(10^−1^), as shown in Figure 4a–d.

**Figure 5:**
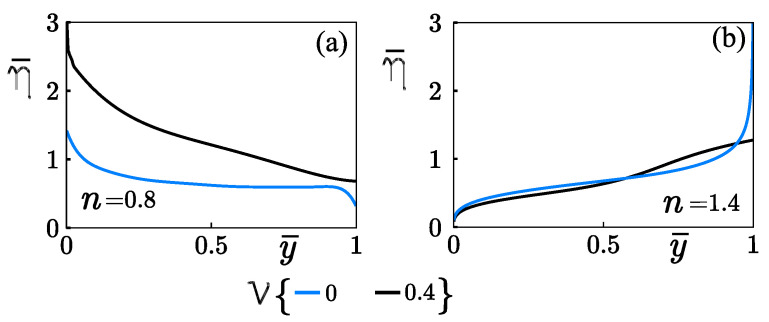
Variation of dynamic viscosity across the microchannel with and without the influence of steric effects. (**a**) Shear-thinning and (**b**) shear-thickening fluids.

**Figure 6:**
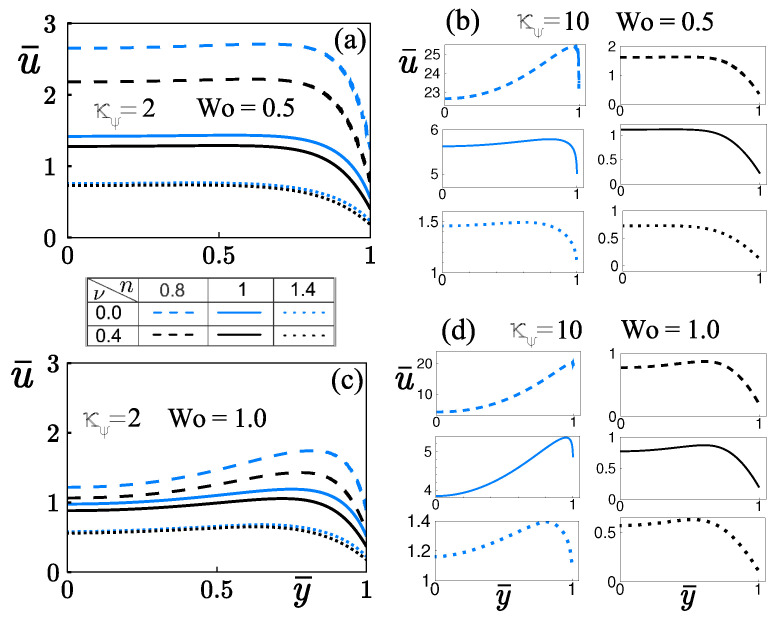
The velocity profiles are shown for shear-thinning and shear-thickening fluids. (**a**): $\kappa_{\psi }=2$, and Wo=0.5. (**b**): $\kappa_{\psi }=10$, and Wo=0.5. (**c**): $\kappa_{\psi }=2$, and Wo=1.0. (**d**): $\kappa_{\psi }=10$, and Wo=1.0.

**Figure 7:**
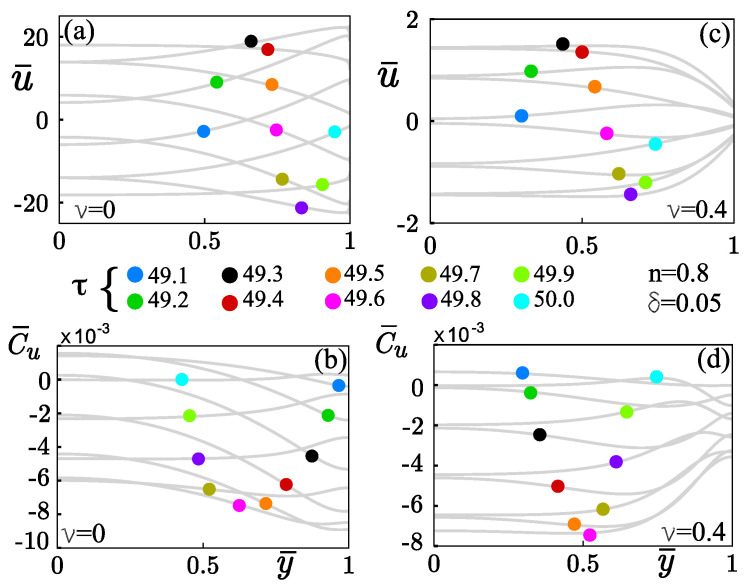
(**a**,**c**): Velocity profiles $\bar{u}$ across the microchannel at one period of oscillation. (**b**,**d**): Distribution of the convective concentration ${\bar{c}}_{u}$ across the microchannel due to the hydrodynamic in (**a**,**c**), respectively.

**Figure 8:**
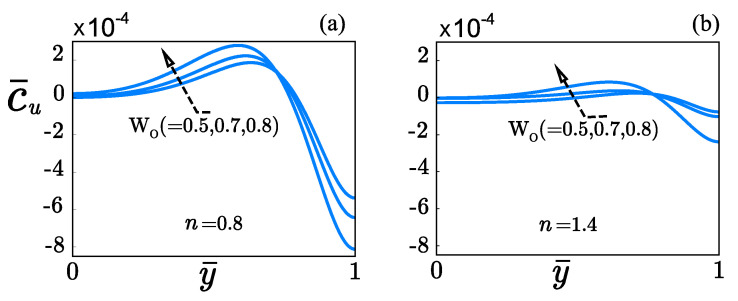
Distribution of dimensionless convective concentration ${\bar{c}}_{u}$ across the microchanne at different Womersley numbers Wo. (**a**) Shear-thinning fluids. (**b**) Shear-thickening fluids.

**Figure 9:**
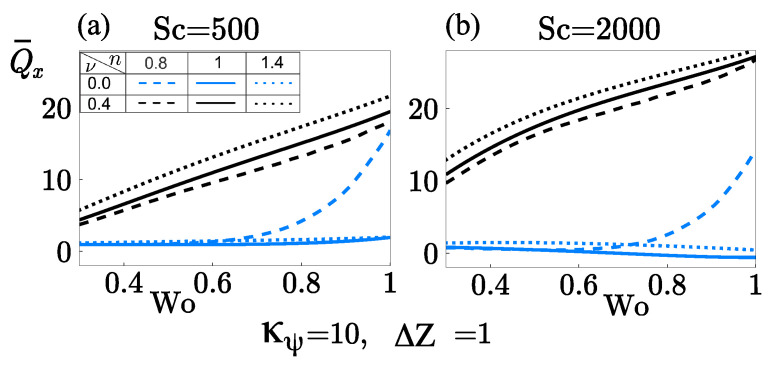
Rate of mass transport ${\bar{Q}}_{x}$ as a function of Wo at different flow behavior indices *n* and the steric factor ν. (**a**) Sc=500 and (**b**) Sc=2000.

**Figure 10:**
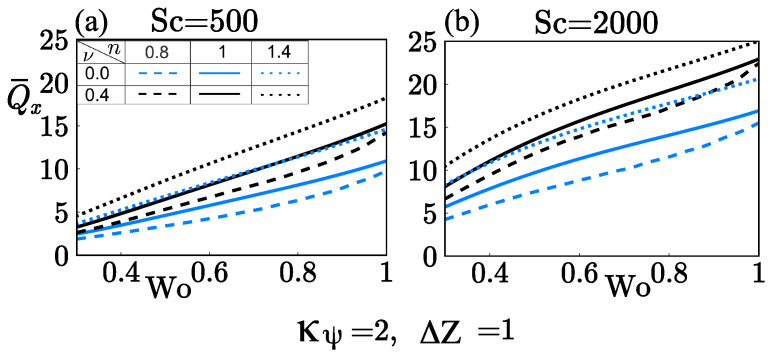
Rate of mass transport ${\bar{Q}}_{x}$ as a function of Wo at different flow behavior indices *n* and the steric factor ν. (**a**) Sc=500 and (**b**) Sc=2000.

**Figure 11:**
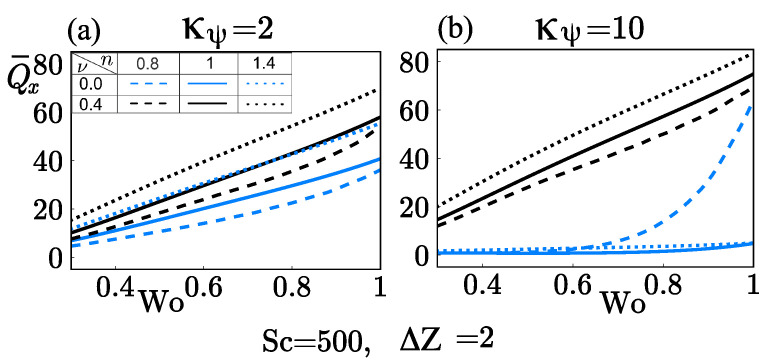
Rate of mass transport ${\bar{Q}}_{x}$ as a function of Wo at different flow behavior indices *n* and the steric factor ν. (**a**) $\kappa_{\psi }=2$ and (**b**) $\kappa_{\psi }=10$.

**Figure 12:**
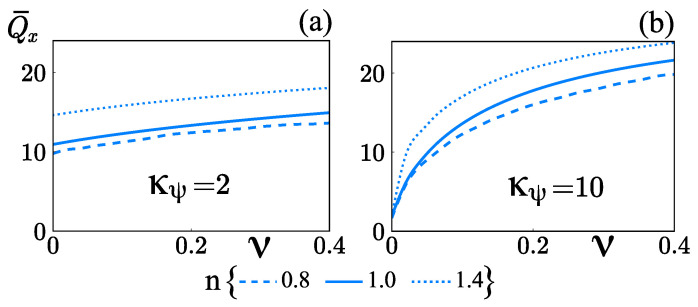
Rate of mass transport ${\bar{Q}}_{x}$ as a function of the steric effect factor ν, at different flow behavior indices *n*. (**a**) $\kappa_{\psi }=2$ and (**b**) $\kappa_{\psi }=10$.

As the dimensionless time τ goes on, the flow begins a state of damping; the amplitude begins by growing in this way but eventually settles down to a constant value, acquiring an oscillatory and periodic state. Figure 4a,b show the cases when the slippage is absence (δ=0), the velocity decreases with the finite-sized ions (ν) in Newtonian and non-Newtonian flows. In Figure 4c,d shows the effect of the slippage (δ=0.05), and the velocity is reduced up to one order of magnitude by considering the finite-sized ions (ν=0.4). That reduction in the velocity is attributed to the fact that the finite ionic volumes give rise to the reduction of the ionic concentration inside EDL, therefore decreasing the oscillatory electroosmotic body force. Hydrophobic condition promotes the increment in the flow velocity affected by the steric effect, as shown in Figure 4b,d. It is illustrated in Figure 4d, with δ=0.05, the dimensionless velocity at τ∼O(10^1^) when the periodic state is reached, $\bar{u}\approx 1.4$, representing an increase of approximately 75% with respect to the no-slippage case, where $\bar{u}\approx 0.8$, as is shown in Figure 4b. In shear-thinning fluids (Figure 4a,c), due to the shear-rate dependent viscosity, the start-up transient motion will die out in a faster dimensionless time $\tau ∼O(10^{0})$ because of the viscosity decreases (see Figure 5a, blue curve) when the shear rate increases. That is due to the ions are treated as electric charges having no volume into the EDL, increasing the electroosmotic body force and consequently modify the shear rate of the fluid. Conversely, the finite-sized ions in shear-thinning fluids delayed the transient state to tend to its periodic-state in a dimensional time of order ∼$O(10^{1})$, as shown in Figure 4b,d. In Newtonian and shear-thickening fluids, the transient state will tend to its periodic-state much faster than shear-thinning fluids in a dimensional time of order ∼$O(10^{-1})$, as shown in all the panels of Figure 4. Therefore, this paper is mainly focused on the transport of neutral solutes in the periodic -state. A dimensionless time of order τ=40 was selected to ensure all initial transients have died out in the flow. The hydrodynamic and the concentration of species presented in Figure 5, Figure 6, Figure 7 and Figure 8, and the rate of mass transport shown in Figure 9, Figure 10, Figure 11 and Figure 12, are all referred to the periodic state of the OEOF.

Figure 5 shows the influence of the finite-sized ions in the fluid’s rheology by considering the hydrophobic condition. Both Figure 5a,b were determined with δ=0.05, Wo=1, $\bar{\kappa }=10$, $\kappa_{\psi }=10$, and τ=49.4. In Figure 5a (shear-thinning fluids), the absence of the steric effect gives rise to a non-linear variation of the dynamic viscosity across the microchannel given in Equation (16), and it decreases from the centerline up to the wall. The steric effect’s presence promotes higher values of dynamic viscosity than when the steric effect is absent. That increment in the dynamic viscosity is due to the finite-sized ions reducing the ion-concentration into the EDL and, consequently, the electric body force, offering the fluid greater resistance to deformed. In shear-thickening fluids, dynamic viscosity increases from the centerline up to the wall with and without steric effects, as is shown in Figure 5b. The dynamic viscosity near walls by considering ν=0 is higher than that obtained with ν=0.4; this occurs due to velocity gradients strongly affected with finite-sized ions be discussed later in Figure 6b,d. Interestingly, it is observed that, in specified values of δ=0.05, Wo=1, $\bar{\kappa }=10$, $\kappa_{\psi }=10$, τ=49.4, the fluid acts as an inviscid flow at the centerline for the special case of n=1.4.

In Figure 6, the influence of $\kappa_{\psi }=2$, and high zeta potentials ($\kappa_{\psi }=10$), with two different values of the Womersley number on the velocity profiles $\bar{u}$, as a function of the coordinate $\bar{y}$ for shear-thinning (n=0.8), Newtonian (n=1), and shear-thickening fluids (n=1.2) are plotted. All panels were determined with δ=0.05, $\bar{\kappa }=10$, and τ=49.2. Dashed, solid, and dotted lines correspond to shear-thinning, Newtonian and shear-thickening fluids, respectively. Curves in blue do not consider the finite-sized ions (steric effects), and black curves consider the steric effects. In Figure 6a, the steric effect promotes a decrease in non-Newtonian and Newtonian fluids’ velocity; that behavior is more representative in shear-thinning fluids. This reduction in hydrodynamics can be avoided and can even be exceeded by applying high zeta potentials of the order of $\kappa_{\psi }∼O\left(10^{1}\right)$, keeping fixed Wo, as shown in Figure 6b. It is evident from Figure 6a,b that the velocity increases in one order of magnitude by increasing $\kappa_{\psi }$ from 2 to 10; however, with $\kappa_{\psi }=10$ (Figure 6b), the velocity’s reduction by steric effects in Newtonian and Non-Newtonian fluids is more significant than that obtained by considering $\kappa_{\psi }=2$. That occurs because the body electric forced is reduced by the finite-sized ions into the EDL; this causes a significant decrement in the slippage velocity at the walls changing the velocity profiles from concave to convex shape (Figure 6b,d). In addition, steric effects give rise to a strong reduction in the velocity gradients near the microchannel walls offering the fluid a high resistance to be deformed. The rheology of the power-law fluids plays an important role in reducing velocity gradients due to the dynamic viscosity increases with the steric effect being more pronounced this behavior in shear-thinning fluids, as shown in Figure 5a.

According to Equation (16) and confirmed in Figure 5, in shear-thinning fluids (n<1), the dynamic viscosity is infinite at channel center due to the absence of velocity gradient, as shown in Figure 6a–d, and decreases at channel wall where higher velocity gradients are reached, while the opposite is true for shear-thickenings (n>1). In this context, the shear-thinning behavior is more remarkable when Wo increases from 0.5 to 1, as depicted in Figure 6a,c, maintaining fixed the zeta potential $\kappa_{\psi }=2$. For shear-thickening fluids (Figure 6c), the steric effect does not modify the velocity profiles, becoming a little concave in the center of the channel, and the important changes in velocity occur in the neighborhood of the sidewalls, as shown in Figure 6a,c. In Figure 6d, steric effects in Newtonian and power-law fluids provoke a representative decrease in the velocity on the microchannel cross-section. That reduction in velocity is more significant in shear-thinning fluids in one order of magnitude in comparison when the steric effects are absent. In addition, steric effects modified the shape of the velocity profiles from concave to convex due to the rheology of power-law fluids explained in Figure 5.

The combined effects of the hydrophobic surface condition and the finite-sized ions on the velocity and species concentration fields are shown in Figure 7. All panels were determined with n=0.8, Wo=1, $\bar{\kappa }=10$, $\kappa_{\psi }=10$, δ=0.05, Sc=500, α=0.001. Because a pure AC electric field drives the electroosmotic flow, the movement of the flow acquires an oscillatory behavior, as shown in Figure 7a,c, and, consequently, the corresponding concentration field, as depicted in Figure 7b,d. The velocity $\bar{u}$ is plotted over one period of oscillation τ (=49.1–50) as a function of the transversal coordinate $\bar{y}$, for shear-thinning fluids with n=0.8, Wo=1, $\bar{\kappa }=10$, $\kappa_{\psi }=10$, Sc=500, and α=0.001, by considering a dimensionless Navier slip length, δ=0.05. When the ions are treated as point charges, i.e., ν=0, the high zeta-potential $\kappa_{\psi }=10$ magnifies the slippage effect on the hydrodynamics of the OEOF, as indicated in Figure 7a. That is because, in the absence of the ionic size effects, shear-thinning fluids (n<1) exhibit smaller viscosity at the wall than that with steric effects, as pointed in Figure 5a. That explains why the fluid has a low resistance to be deformed, causing high-velocity gradients in the walls’ vicinity. According to the oscillatory electroosmotic body force included in Equation (15),

(41) $${\bar{\rho }}_{e}{\bar{E}}_{x}\left(\tau \right)=\frac{{\bar{\kappa }}^{2}}{\kappa_{\psi }}\frac{\sinh\bar{\psi }}{1+2\nu \sinh^{2}\left(\bar{\psi }/2\right)}\sin\left(2\pi \tau \right),$$

when the steric effect is absent (ν=0) in Equation (41), and considering fixed values in $\bar{\kappa }$ and $\bar{\psi }=\kappa_{\psi }$, the electric body force at the wall, where *A* is a constant, ${\bar{\rho }}_{e}{\bar{E}}_{x}∼A\sin\left(2\pi \tau \right)$ promotes the highest velocity gradients, namely for half-period τ (=49.1–49.4) the flow moves in the positive axial direction and the body force Asin(2πτ)>0, while, for τ (=49.6–49.9), the flow moves in the opposite direction and the function Asin(2πτ)<0; in both scenarios described before, the velocity profiles acquire concave shape in the flow direction. At specific times τ (=49.5 and 50), the electric body force Asin(2πτ)=0, causing a significant decrease in velocity and velocity gradients. Then, velocity profiles acquired a convex shape, and the velocity is close to zero in the neighborhood of the sidewalls, as is shown in Figure 7a. On the other hand, in Figure 7c, the finite-sized ions (ν≠0) affect the slippage condition at the wall, giving rise to a reduction in the velocity up to one order of magnitude, and consequently, the velocity gradients are smaller than that obtained with ν=0. That reduction in velocity gradients is attributed to reducing the ionic concentration inside EDL as stated in Equation (3), decreasing the oscillatory electroosmotic body force in Equation (41). For example, an estimation of ${\bar{\rho }}_{e}{\bar{E}}_{x}{\left(\tau \right)|}_{\bar{y}=1}$ near the microchannel-wall, where $\bar{\kappa }∼O\left(10^{1}\right)$, $\kappa_{\psi }∼O\left(10^{1}\right)$, and $\bar{\psi }∼\kappa_{\psi }$; for a specific τ=49.2, when ν=0 the electric body force ${\bar{\rho }}_{e}{\bar{E}}_{x}{\left(\tau \right)|}_{\bar{y}=1}∼O\left(10^{4}\right)$, and when steric effects are considered with ν=0.4, the electric force ${\bar{\rho }}_{e}{\bar{E}}_{x}{\left(\tau \right)|}_{\bar{y}=1}∼O\left(10^{1}\right)$.

The dimensionless concentration ${\bar{c}}_{u}$ plotted in Figure 7b,d corresponds to the velocity field $\bar{u}$ in igure Figure 7a,c over the same period of oscillation. Figure 7b shows the concentration, ${\bar{c}}_{u}$, without steric effects. In the first half period of oscillation τ (=49.1–49.6), the concave flow profile causes transversal concentration gradients, and the neutral dilute solute diffuses from the centerline of the microchannel to lateral walls. During the second half interval of time τ (=49.7–50), the flow profile is reversed, and the solute moves from the boundary walls to the centerline, where the solute’s concentration is small compared to its value at the neighborhood of the walls. Figure 7d shows the concentration distribution with the steric effect. Due to a strong decrease in the slip-velocity at the walls originated by steric effects, the values of ${\bar{c}}_{u}$ near the walls are small compared to its value at the centerline; therefore, neutral dilute solute diffuses from the lateral walls to the centerline of the microchannel.

The rheology plays an essential role on the distribution of the concentration of the solute ${\bar{c}}_{u}$, as shown in Figure 8. For instance, higher concentration gradients across the microchannel-width appear with shear-thinning fluids (Figure 8a) in comparison against those present in shear-thickening fluids (Figure 8b). It occurs when *Wo* increases from 0.5 to 0.8, keeping fixed ν=0.4, $\kappa_{\psi }=10$, $\bar{\kappa }=10$, Sc=500, ΔZ=1, δ=0.05, and α=0.001. In Figure 8a, the distribution in ${\bar{c}}_{u}$ is more dissimilar as *Wo* increases. This consequence arises from the non-uniformity in the velocity flow becoming stronger in shear-thinning fluids, as shown in Figure 6d. The concentration ${\bar{c}}_{u}$ in shear-thickening fluids is less affected by the Womersley number *Wo* as presented in Figure 8b in comparison with shear-thinning fluids, due to a significant resistance offering by the fluid to be deformed, as shown in Figure 6d, where the velocity is reduced by about 30% concerning that obtained in shear-thinning fluids under the influence of steric effects.

Oscillatory flows become a subject of considerable interest due to their potential to provide significant advantages in the manipulation of the transport of species, causing that those with low diffusivities can be transported faster than species with higher diffusivities, and vice versa. In this context, the rate of mass transport ${\bar{Q}}_{x}$ is shown in Figure 9a,b as a function of the Womersley number Wo with ${\bar{\kappa }}_{\psi }=10$, $\bar{\kappa }=10$, δ=0.05, α=0.001 and ΔZ=1. Dashed, solid, and dotted lines correspond to shear-thinning, Newtonian and shear-thickening fluids, respectively. Curves in blue do not consider the finite-sized ions (steric effects), and black curves consider the steric effects. Figure 9a analyzes a fast diffuser (Sc=500), while Figure 9b analyzes a slow diffuser (Sc=2000).

The mass transport ${\bar{Q}}_{x}$, in a fast diffuser in the absence of steric effects, Wo does not influence on ${\bar{Q}}_{x}$ in the interval 0.3≲Wo≲0.6 in fluids with *n* (=0.8, 1.0, 1.4), as indicated in Figure 9a. Shear-thinning fluids with Wo≳0.6 promote a rapid increase in ${\bar{Q}}_{x}$ while Newtonian and shear-thickening fluids remain unaltered with Wo. That occurs in fluids with n<1 because of high slippage velocity at the walls and the concave shape of velocity profiles in the flow direction with one order of magnitude higher than fluids with n=(1,1.4) where the velocity profiles acquire flattened shape, as shown in blue curves in Figure 6b. Conversely, in the presence of steric effects, Wo enhances ${\bar{Q}}_{x}$ in fluids with *n* (=0.8, 1.0, 1.4), as indicated by black-curves in Figure 9a. It is observed that independently of the value that Wo takes, the convex-shape (black lines, in Figure 6b,d) of the velocity profiles in fluids with *n* (=0.8, 1.0, 1.4) remain in the same order of magnitude. However, the shear-thickening fluid is a candidate to transport more species due to shear-thinning fluids offer greater resistance to being deformed in the presence of steric effects, as it is demonstrated in Figure 5a. Figure 9b shows how the importance of the hydrodynamic behavior mentioned above in benefiting and enhance the mass transport of the slow diffuser (Sc=2000) with a shear-thickening as a carrier fluid in the presence of steric effects. That is, species with low diffusivity (Sc=2000) can be transported up to 64.6% faster than species with high diffusivity (Sc=500) when the steric effect is considered. Contrarily, when the steric effect is neglected, the more diffusive species travel slightly faster up to 21.5%.

Figure 10 highlights the effect of the zeta potential ${\bar{\kappa }}_{\psi }=2$ on ${\bar{Q}}_{x}$, with $\bar{\kappa }=10$, δ=0.05, α=0.001, and ΔZ=1. As expected, the zeta potential attenuates the mass transport of fast and slow diffusers with shear-thickening fluid as carrier compared to high-zeta potential, as described in Figure 9. However, the zeta potential improves ${\bar{Q}}_{x}$ with shear-thinning fluids, as shown in Figure 10. In addition, the steric effect enhances the mass transport in fast and slow diffusers when Wo increases. The reason is that independently the value Wo takes, the reduction in velocity by steric effects remains in the same order of magnitude, except in shear-thickening fluids where steric effects are tiny, as shown in Figure 6a,b.

Furthermore, Figure 10a and Figure 11a were compared to analyze the influence of tidal displacement ΔZ on the mass transport rate ${\bar{Q}}_{x}$. A tidal displacement ΔZ=2 causes an increment in the mass transport rate about two times with ${\bar{\kappa }}_{\psi }=2$ compared to that obtained with ΔZ=1, and that increment was estimated up to four times higher when high zeta potentials (${\bar{\kappa }}_{\psi }=2$) were considered (see Figure 9a and Figure 11b). From Equation (31), it is evident that the tidal displacement influences the concentration ${\bar{c}}_{u}$ through $Pe_{\omega }$; as can be deduced from Equation (39), ${\bar{c}}_{u}$ will affect the mass transport rate, ${\bar{Q}}_{x}$. Then, the tidal displacement increases the convective effects in the concentration field and enhances the mass transport rate in comparison when a smaller tidal displacement is used.

Finally, the dependency of the rate of mass transport ${\bar{Q}}_{x}$ on the steric factor is studied and Figure 12 displays the results with $\bar{\kappa }=10$, δ=0.05, α=0.001, Sc=500, ΔZ=1, and Wo=1. When the ions are assumed to have finite volumes, the mass transport in power-law and Newtonian fluids is an ascending function of the steric factor, ν, for ${\bar{\kappa }}_{\psi }=2$, and high $\left({\bar{\kappa }}_{\psi }=10\right)$ zeta potentials, as it is shown in Figure 12a,b. In Figure 12a, the values of ${\bar{Q}}_{x}$ for ν=0 and ν=0.4 with the power-law index *n* (=0.8, 1.0, 1.4) correspond to the values of ${\bar{Q}}_{x}$ in Figure 10a with Wo=1. In Figure 12a, the mass transport ${\bar{Q}}_{x}$ in shear-thickening fluids is higher than in Newtonian and shear-thinning fluids. This is due to in shear-thickening fluids there is a little influence of the steric factor on the velocity profiles, while, for Newtonian and shear-thinning fluids the velocity profiles are reduced by steric effects, as shown in Figure 6c. This occurs because, fluids with n>1 offer more resistance to be deformed in comparison with Newtonian and shear-thinning fluids. This is confirmed in Figure 5, the steric effect in fluids with n=1.4 has a little impact on the apparent viscosity (see Figure 5b), while, in fluids with n=0.8, an important increment in the viscosity distribution occurs due to steric effects, as shown in Figure 5a.

In Figure 12b, the values of ${\bar{Q}}_{x}$ as a function of ν with the power-law index *n* (=0.8, 1.0, 1.4) and $\kappa_{\psi }=10$ are plotted. The increase of ${\bar{Q}}_{x}$ with ν is more pronounced with $\kappa_{\psi }=10$ than with $\kappa_{\psi }=2$. That is due to near the wall, the reduction of the body force due to steric effects is significant; consequently, a higher zeta potential gives rise to a smaller velocity, as it is shown in Figure 6d, where for fluids with n=0.8, the velocity is reduced in one order of magnitude, and fluids with n=1.0 and n=1.4, the reduction in the velocity are maintained in the same order of magnitude.

## 5. Conclusions

The effects of non-Newtonian rheology, steric effect, and slippage on an OEOF were analyzed. The start-up condition for the flow was considered, and after all initial transients have died out and the flow is periodic, the mass concentration and transport of species were determined numerically. The influence of the main parameters that describe the dynamic behavior of the flow is the steric factor ν, the normalized slip length, δ, an electrokinetic parameter $\bar{\kappa }$, the normalized zeta potential $\kappa_{\psi }$, the Womersley (Wo) and Schmidt (Sc) numbers, and the power-law index *n*. The ionic size effects were controlled by the mean volume fraction of each ion in bulk given by the factor $\nu =2a^{3}n_{0}$. The numerical results are compared against an OEOF solution for a Newtonian fluid without slippage, and steric effects, previously reported by H. Huang and C. Lai [13]. The following conclusions from this study were drawn:

1. In shear-thinning fluids, the steric effect under hydrophobic conditions has a noticeable impact on the rheology of the fluid, causing higher values in the dynamic viscosity, $\bar{\eta }$, compared with the absence of finite-size ions. In shear-thickening fluids, since steric effects reduce the oscillatory electroosmotic body force up to three orders of magnitude compared with no steric case, the dynamic viscosity decreases near the microchannel wall.
2. Finite-size ions reduce oscillatory electroosmotic body force by preventing EDL from being highly concentrated. As a result, steric effects result in a decrease in velocity in shear-thinning fluids up to one order of magnitude compared with no steric case, with $\kappa_{\psi }=10$. However, in shear-thickening fluids, the steric effects are negligible on the velocity when $\kappa_{\psi }=2$.
3. The suggested values of $Wo∼O(10^{-1})$, $\kappa_{\psi }=10$ and ν=0.4 promote the best conditions for the mass transport ${\bar{Q}}_{x}$ for any Sc number values. A value of ν=0.4 with $\kappa_{\psi }=10$ increases the value of ${\bar{Q}}_{x}$ in about 90 % compared with no steric effect. In a similar way, in about 20 % the value of ${\bar{Q}}_{x}$ was increased with $\kappa_{\psi }=2$.
4. The steric effect enhances the mass transport in fast and slow diffusers when Wo increases by using ${\bar{\kappa }}_{\psi }=2$ or high zeta potentials (${\bar{\kappa }}_{\psi }=10$). However, at high zeta potentials (${\bar{\kappa }}_{\psi }=10$), ${\bar{Q}}_{x}$ increases up to one order of magnitude compared with that obtained with ${\bar{\kappa }}_{\psi }=2$. Additionally, steric effect promotes that slow diffusers (Sc=2000) can travel faster than fast diffusers (Sc=500) at Wo<1, as shown in Figure 9 (black lines). The opposite behavior occurs in the absence of steric effects.
5. A wide variety of different physical and chemical phenomena could also be included in the model to examine their effects on mass transport with non-Newtonian fluids. Possibilities include the depletion of macromolecules near the microchannel walls, the presence of a reversible reaction or mass exchange between the microchannel wall and the fluid. In physiological systems, which can be significantly more important viscoelastic effects than higher purity for typical aqueous solutions, could be considered.

## Figures and Tables

**Table 1:** Order of magnitude of the dimensionless quantities.

Dimensionless Quantities	Definition	Order of Magnitude
Sc	Schmidt number, $\left(\nu_{0}/D\right)$	∼$O(10^{2}$–$10^{3})$
Wo	Womersley number, $\left(h\sqrt{\omega /\nu_{0}}\right)$	∼O(1)
α	Aspect ratio, (h/L)	∼$O(10^{-3})$
$\bar{m}$	Consistency index, $n\left(\frac{m}{\mu }\right){\left(\frac{U_{HS}}{h}\right)}^{n-1}$	∼$O(10^{-1}$–$10^{0})$
ΔZ	Tidal displacement, (Δz/h)	∼O(1)
δ	Slip lenght, $\left(\lambda_{N}/h\right)$	∼0.05
$\kappa_{\psi }$	Potential ratio, $\left(ze\zeta /k_{B}T\right)$	∼2,10
$\bar{\kappa }$	Electrokinetic parameter, (κh)	∼10,20
ν	Steric factor, ($2a^{3}n_{0}$)	∼O(0–0.4)

**Table 2:** Physical properties and geometrical parameters used for estimating the dimensionless parameters from the present analysis.

Parameter	Definition	Value
*a*	Ion size	∼2 nm [15]
$c_{0}$	molar concentration	∼(50–100) mol m^−3^ [15]
*D*	Diffusion coefficient	∼$(10^{-9}$–$10^{-8})$ m^2^ s^−1^ [45]
*e*	Electron charge	∼$1.602\;\times \;10^{-19}$C *
$E_{0}$	Electric field	∼$10^{3}$ V/m [46]
*h*	Microchannel half-height	∼(5–100) m *
$k_{B}$	Boltzmann constant	∼$1.38\;\times \;10^{-23}$ J K^−1^ *
*L*	Micro-channel length	∼$10^{-2}$ m
*m*	Consistency index	∼$(10^{-3}$–$10^{-4}$) Pa s^*n*^ [47]
*n*	Power-law index	(0.8, 1, 1.4) [42]
$n_{0}$	Ionic concentration	∼$10^{25}$ m^−3^ [15]
$N_{A}$	Avogadro number	∼$6.022\;\times \;10^{23}$ mol^−1^ *
*T*	Absolute temperature	∼298 K *
ϵ	Permittivity of the solution	6.95 × $10^{-10}$ C^2^N^−1^m^−2^ *
ζ	Zeta potential	∼(50–260) mV [48]
$\kappa^{-1}$	Debye length	∼(15–300) nm *
$\lambda_{N}$	Navier length	∼$(10^{-9}$–$10^{-6})$ m [49]
μ	Newtonian viscosity	∼$10^{-3}$ Pa s *
$\rho_{f}$	Fluid density	∼$10^{3}$ kg m^−3^ *
ω	Angular frequency	∼400 Hz–5 kHz [50]

* Values taken from Reference [34].

## Abbreviations

*a*	Ion size, m
*c*	Concentration field of the solute, mol m^−3^
$c_{0}$	Molar ion concentration of the electrolyte solution, mol m^−3^
$C_{1},C_{2}$	Fixed concentration values at the ends of the microchannel, mol m^−3^
$c_{u}$	Convective species concentration, mol m^−3^
${\bar{c}}_{u}$	Dimensionless convective species concentration
*D*	Diffusion coefficient, m^2^ s^−1^
D	Rate of strain tensor
*e*	Electron charge, C
$E_{x}\left(t\right)$	External electric field, Vm^−1^
$E_{0}$	Intensity of the applied electric field, Vm^−1^
*h*	Microchannel Half-Height, m
$j_{x,conv}$	Convective flux density, mol m^2^ s^−1^
$j_{x,diff}$	Diffusive flux density *x*, mol m^2^ s^−1^
$J_{x}$	Total flux density, mol m^2^ s^−1^
$K_{B}$	Boltzmann constant, J K^−1^
*L*	Microchannel length, m
*m*	Fluid consistency index, Pa s^*n*^
$\bar{m}$	Dimensionless consistency index
*n*	Power-law index
n	Unit vector normal to the microchannel surface
$n_{0}$	Ionic concentration, m^−3^
$Pe_{D}$	Diffusive Péclet number
$Q_{x}$	Flow rate, mol m^−2^ s^−1^
${\bar{Q}}_{x}$	Dimensionless flow rate
Sc	Schmidt number
*t*	Time, s
$t_{\omega }$	Periodic time, s
*T*	Absolute temperature, K
*u*	Longitudinal velocity component, m s^−1^
$u_{s}$	Fluid velocity at the microchannel wall, m s^−1^
$U_{HS}$	Helmholtz-Smoluchowsky velocity
$\bar{u}$	Dimensionless longitudinal velocity component
$W_{o}$	Womersley number
x,y	Space coordinates, m
$\bar{x},\bar{y}$	Dimensionless space coordinates
*z*	valency of both the ions
Greek symbols	
α	Aspect ratio
$\dot{\gamma }$	Strain rate
Δz	Tidal displacement, m
ΔZ	Dimensionless tidal displacement
δ	Dimensionless slip lenght
ϵ	Permittivity of the solution, C V^−1^ m^−1^
ζ	Zeta potential, V
η	Apparent viscosity of the fluid, Pa s
$\kappa^{-1}$	Debye length, m
$\kappa_{\psi }$	Relation between the wall ζ potential and the thermal potential
$\bar{\kappa }$	Ratio of the microchannel height to the Debye length
$\lambda_{N}$	Navier length, m
μ	Viscosity of a Newtonian fluid, Pa s
ν	Bulk volume fraction of ions
$\upsilon_{0}$	Kinematic viscosity, m^2^ s^−1^
$\rho_{f}$	Fluid density, kg m^−3^
$\rho_{e}$	Electric charge density, C m^−3^
τ	Dimensionless time
$\tau_{\omega }$	Dimensionless periodic time
$\tau_{xy}$	Unidirectional shear stress
Φ	Total electric potential, V
ϕ	External electric potential, V
ψ	Electric potential, V
ω	Angular frequency, rad s^−1^
Subscripts and superscripts	
*i*	Nodal position in $\bar{x}$ coordinate
*j*	Nodal position in $\bar{y}$ coordinate
*l*	Nodal position in time
tr	Matrix transpose
Abbreviations	
AC	Alternating current
DC	Direct current
DNA	Deoxyribonucleic acid
EDL	Electrical double layer
EOF	Electroosmotic flow
MPB	Modified Poisson-Boltzmann
OEOF	Oscillatory electroosmotic flow
PB	Poisson-Boltzmann
TDMA	Tridiagonal matrix algorithm
